# Evaluation of objective methods for analyzing ipsilateral motor evoked potentials in stroke survivors with chronic upper extremity motor impairment

**DOI:** 10.1088/1741-2552/ada827

**Published:** 2025-04-22

**Authors:** Akhil Mohan, Xin Li, Bei Zhang, Jayme S Knutson, Morgan Widina, Xiaofeng Wang, Ken Uchino, Ela B Plow, David A Cunningham

**Affiliations:** 1 Department of Biomedical Engineering, Lerner Research Institute, Cleveland Clinic, Cleveland, OH, United States of America; 2 Department of Biomedical Engineering, Case Western Reserve University, Cleveland, OH, United States of America; 3 Department of Physical Medicine and Rehabilitation, The MetroHealth System, Case Western Reserve University School of Medicine, Cleveland, OH, United States of America; 4 Cleveland Functional Electrical Stimulation Center, Cleveland, OH, United States of America; 5 Department of Quantitative Health Sciences, Lerner Research Institute, Cleveland Clinic, Cleveland, OH, United States of America; 6 Cerebrovascular Center, Neurological Institute, Cleveland Clinic, Cleveland, OH, United States of America; 7 Department of Physical Medicine and Rehabilitation, Neurological Institute, Cleveland Clinic, Cleveland, OH, United States of America

**Keywords:** stroke, motor evoked potential, transcranial magnetic stimulation, ipsilateral pathways, reliability

## Abstract

*Objective.* Ipsilateral motor evoked potentials (iMEPs) are believed to represent cortically evoked excitability of uncrossed brainstem-mediated pathways. In the event of extensive injury to (crossed) corticospinal pathways, which can occur following a stroke, uncrossed ipsilateral pathways may serve as an alternate resource to support the recovery of the paretic limb. However, iMEPs, even in neurally intact people, can be small, infrequent, and noisy, so discerning them in stroke survivors is very challenging. This study aimed to investigate the inter-rater reliability of iMEP features (presence/absence, amplitude, area, onset, and offset) to evaluate the reliability of existing methods for objectively analyzing iMEPs in stroke survivors with chronic upper extremity (UE) motor impairment. *Approach.* Two investigators subjectively measured iMEP features from thirty-two stroke participants with chronic UE motor impairment. Six objective methods based on standard deviation (SD) and mean consecutive differences (MCD) were used to measure the iMEP features from the same 32 participants. IMEP analysis used both trial-by-trial (individual signal) and average-signal analysis approaches. Inter-rater reliability of iMEP features and agreement between the subjective and objective methods were analyzed (percent agreement-PA and intraclass correlation coefficient-ICC). *Main results.* Inter-rater reliability was excellent for iMEP detection (PA > 85%), amplitude, and area (ICC > 0.9). Of the six objective methods we tested, the 1SD method was most appropriate for identifying and analyzing iMEP amplitude and area (ICC > 0.9) in both trial-by-trial and average signal analysis approaches. None of the objective methods were reliable for analyzing iMEP onset and offset. Results also support using the average-signal analysis approach over the trial-by-trial analysis approach, as it offers excellent reliability for iMEP analysis in stroke survivors with chronic UE motor impairment. *Significance.* Findings from our study have relevance for understanding the role of ipsilateral pathways that typically survive unilateral severe white matter injury in people with stroke.

## 1. Introduction

Transcranial magnetic stimulation (TMS) is a non-invasive brain stimulation technique for evaluating the strength of corticospinal pathways originating from the human motor cortex. Evoked motor output is typically recorded from muscles in the contralateral (opposite) limb using surface electromyography (EMG), known commonly as contralateral motor evoked potentials (cMEPs). CMEPs represent the excitability of monosynaptic, crossed, corticospinal pathways from the contralateral motor cortex [1, 2]. Features such as size and latency signify strength and conduction velocity respectively of the corticospinal transmission [3].

Evoked motor output can also be recorded from muscles in the ipsilateral (same) limb, and are known as ipsilateral motor evoked potentials (iMEPs). IMEPs are believed to represent cortically-evoked excitability of uncrossed brainstem-mediated pathways [4–6]. Typically, these pathways may include the thinly myelinated, polysynaptic reticulospinal, rubrospinal, and vestibulospinal tracts, which have preferential innervation of proximal large flexor muscle groups (e.g. biceps brachii) [4–8]. Ipsilateral pathways are abundant in infancy and early childhood but become masked as neurologic maturity is reached [9]. In the event of extensive injury to (crossed) corticospinal pathways as which can occur following stroke, it is believed that ipsilateral pathways become unmasked to serve as alternate output to muscles of the paretic upper extremity (UE) [10–13].

However, discerning iMEPs are challenging, even in neurally intact people, because they are small compared to cMEPs and not as easily elicited, necessitating volitional activation of the test muscle [6]. Therefore, discerning them is even more challenging in stroke survivors with chronic motor impairment since paretic muscles can only generate weak, inconsistent bursts of volitional activity that make it difficult to distinguish evoked potentials from ongoing activity of the paretic muscle.

There are no widely accepted methods for identifying and analyzing iMEPs. Moreover, most of the reported methods have been evaluated in neurologically healthy adults, where iMEPs can be discerned with greater consistency due to the intact voluntary EMG activity in the muscle [4–6, 14–19]. For example, when analyzing EMG signals evoked by TMS, some studies classify a signal as iMEP (or iMEP+) when the post-TMS signal exceeds the pre-TMS signal by at least 1 standard deviation (SD) for 5 ms [4, 18] or when the post-TMS signal correlates with the post-TMS reference template signal by at least 0.7 [14]. This is known as the trial-by-trial analysis. A few other studies have averaged all EMG signals across several TMS trials and defined the resultant EMG signal as iMEP+ if the averaged rectified post-TMS EMG exceeded pre-TMS EMG signal by 0.1 mV, known as the average-signal analysis [6, 16, 20]. A few studies have also recommended using the mean consecutive difference (MCD) threshold method for analyzing EMG data, as SD methods are not best suited for analyzing dependent observations [21, 22]. The MCD threshold method uses the variability in the pre-TMS EMG compared to the average pre-TMS EMG used in the SD threshold method. Employing varied methodologies (such as trial-by-trial analysis vs. average-signal analysis) and thresholds (SD based vs. MCD-based) for iMEP analysis can increase variability among researchers and laboratories, reduce reproducibility, and make cross-study comparisons challenging. Consequently, there’s a pressing need to assess the reliability of iMEP analysis methods and optimize them for use in stroke survivors with chronic UE motor impairment.

The purpose of this study was to investigate the inter-rater reliability of iMEP features (presence/absence, amplitude, area, onset, and offset) in stroke survivors with chronic UE motor impairment. We further assessed the agreement between subjective (rater-determined) and objective (algorithm-driven) iMEP analysis methods in both trial-by-trial and average-signal analysis approaches. We selected six threshold levels based on SD and MCDs published across several studies to analyze iMEP features in stroke survivors with chronic UE motor impairment [4–7, 18–21, 23–25]. This study seeks to evaluate existing objective algorithms for identifying and analyzing iMEPs in people with stroke. The systematic analysis of objective methods for measuring the integrity and viability of uncrossed, ipsilateral corticomotor pathways is important for improving the quality of UE rehabilitation interventions that target ipsilateral mechanisms for functional recovery.

## 2. Methods/design

### 2.1. Participants

Participants between 18 and 90 years of age who have suffered ischemic or hemorrhagic stroke with chronic (⩾6 months) upper limb paresis were included in the study. Individuals with stroke affecting the cerebellum or brainstem or those with a history of bilateral/multiple strokes affecting sensorimotor function were excluded. Those with severe cognitive impairment (unable to follow 3-step command), severe spasticity (modified Ashworth score = 4), or contracture of the wrist/hand were also excluded. Exclusion criteria related to TMS were presence of any metal in the head, history of seizures, pacemakers, and any medication that could increase seizure threshold (e.g. bupropion).

The study was approved by the institutional review board of the Cleveland Clinic and conformed to the Declaration of Helsinki. All participants provided written informed consent before starting study procedures.

### 2.2. Assessments

#### 2.2.1. Motor impairment assessment

Level of motor impairment was characterized using the UE Fugl-Meyer (UEFM) scale [26]. UEFM tests single- and multiple-joint movements of the shoulder, elbow, wrist and hand in and out of synergy. Each item is graded on a 3-point ordinal scale (0, cannot perform; 1, perform partially; and 2, perform fully) and summed to provide a maximum score of 66. UEFM has excellent reliability, consistency, and validity in chronic stroke [27, 28].

#### 2.2.2. Contralateral and ipsilateral pathway neurophysiology

TMS was used to measure the strength of contralateral and ipsilateral pathways. Single monophasic pulses were delivered using a Magstim 200^2^ device with a figure-of-eight coil (inner loop diameter 7 cm, Magstim Inc., Whitland, Wales, UK). Frameless stereotaxic navigation was used to ensure precision and consistency of coil targeting (Brainsight, Rogue Research Inc., Montreal, Quebec, CA). Surface EMG was collected from bilateral biceps brachii, bilateral extensor digitorum communis (EDC), and non-paretic triceps brachii muscles. EMG signals were sampled at 4000 Hz and online band-pass filtered between 10–2000 Hz (PowerLab 4/35 T, ADInstruments Inc., Colorado Springs, CO, USA). LabChart 8 (ADInstruments Inc., Colorado Springs, CO, USA) software was used to visualize data online and store data for offline analysis.

**Figure 1.**
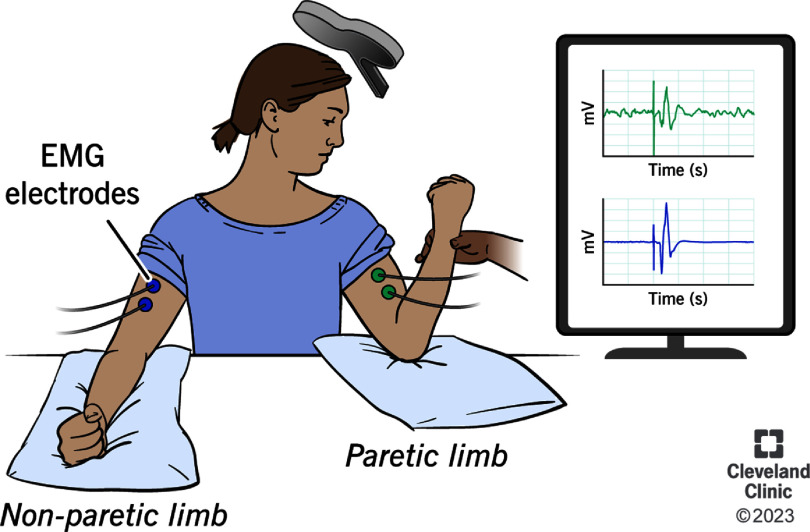
A depiction of the iMEP data collection is shown here. Participants were asked to generate maximal contraction of the paretic biceps and non-paretic triceps with the head rotated towards the paretic side while TMS was delivered to the intact motor hemisphere. Reprinted with permission, Cleveland Clinic Foundation ©2025. All Rights Reserved.

Excitability of crossed corticospinal pathways from the contralateral motor cortex was measured for the paretic and non-paretic EDC muscle, indexed as cMEP. A participant was considered cMEP+ if the post-TMS EMG exceeded the pre-TMS EMG by 0.1 mv or above (peak to peak amplitude) in 6 out of 10 trials; the location of the contralateral motor area at which the lowest TMS intensity produced cMEPs was called as the motor hotspot [29]. Excitability of uncrossed, brainstem-mediated pathways was measured from the paretic biceps brachii muscle by giving supramaximal TMS (100% maximal stimulator output) to the intact hemisphere. We delivered TMS to the EDC motor hotspot and two locations 2 cm anterior and medial to the hotspot, as they were reported to have a greater chance of producing iMEPs [6]. Participants were asked to generate maximal voluntary contraction (100% MVC) of the paretic biceps and non-paretic triceps muscle at the same time, a heteronymous contraction maneuver known to favor the likelihood of eliciting iMEPs (figure 1). Participants were also asked to keep their head turned to the paretic side with neck in slight extension which is believed to activate neck propriospinal afferents that receive terminations of uncrossed brainstem-mediated pathways and thereby increase the likelihood of eliciting iMEPs [6]. We collected 30–40 trials from each participant to maximize the possibility of eliciting 10 identifiable iMEPs.

### 2.3. Data analysis

**Figure 2.**
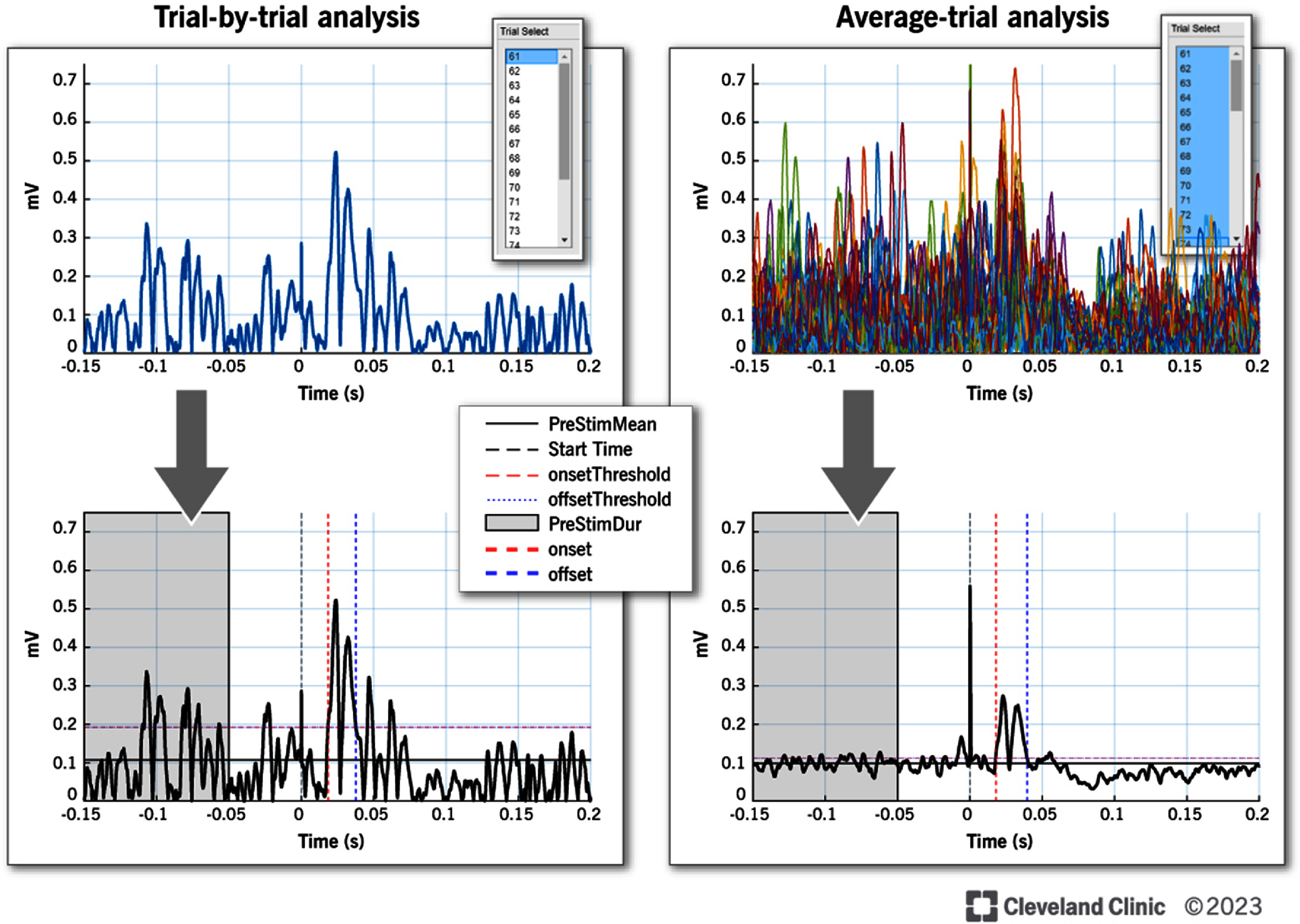
iMEP Analysis using TMS Analysis Toolbox in trial-by-trial and average-signal method are shown here. In the trial-by-trial analysis method, each rectified iMEP trial was analyzed individually for onset, offset, amplitude and area. In the average-signal analysis, the average rectified signal of all 30–40 iMEP trials from a participant was analyzed. Reprinted with permission, Cleveland Clinic Foundation ©2025. All Rights Reserved.

IMEPs were analyzed using subjective and objective methods both in trial-by-trial and average signal analysis approaches (figure 2). In the trial-by-trial analysis method, each rectified iMEP trial was analyzed individually for onset, offset, amplitude and area (details in sections 2.3.1 and 2.3.2). For the average-signal analysis, the average rectified signal of all 30–40 iMEP trials from a participant were analyzed. The iMEP analysis (trial-by-trial and average-signal) was performed offline using the TMS Analysis Toolbox [30]. The TMS Analysis Toolbox has a graphical user interface and allows users to organize large datasets and perform basic and advanced analyses of common TMS related outcomes on individual or averaged signal TMS trials (e.g. MEP latency/amplitudes, silent periods (duration and % decrease), input/output curves (sigmoidal fitting and area under the curve), paired-pulse ratios, and EMG onset detection). Further, it allows interactive analysis for data reduction and outcome detection for immediate visualization and exporting of results for second level analyses. One key feature of the toolbox is that it allows users to import whole multi-channel files from a variety of different data acquisition systems including but not limited to: LabChart, BrainVision, AcqKnowledge, Signal, Spike and Brainsight. As a result, the toolbox can aggregate multiple datasets from diverse research sites which has broad implications for disseminating data.

#### 2.3.1. Subjective analysis

Two investigators (DAC and XL) independently analyzed the iMEP signals in both trial-by-trial and average-signal analysis approaches. Raters used the latencies of biceps cMEP onset and temporal overlay of rectified ipsilateral EMG to classify the EMG signal as valid and invalid iMEPs (iMEP detection). The onset and offset of valid iMEPs were determined by visual inspection [6]. The peak-to-peak amplitude and area of iMEPs within the onset to offset window (iMEP duration) were then measured using the TMS Analysis Toolbox. The subjective analysis can be considered semi-subjective since the iMEP amplitude and area within the iMEP duration were calculated using the TMS Analysis Toolbox. IMEP area was calculated using the following formula [6],

\begin{align*}&amp;{\text{iMEP area}}\!=\! \frac{{{\text{area of rectified EMG in iMEP duration}}}}{{\left( {{\text{mean pre}} -{{\text {TMS EMG}}}} \right)\times\left( {{\text{iMEP duration}}} \right)}}\!\times\!{{100}}\end{align*}

The mean pre-TMS EMG was measured 100 ms before the TMS artifact.

#### 2.3.2. Objective analysis

The same EMG signals were analyzed using signal processing algorithms. The analysis algorithm compared post-TMS EMG to threshold level defined as pre-TMS EMG + X* SD (where *X* = 0, 1, 2, and 3) and pre-TMS EMG + *Y* * MCD (where *Y* = 1.77 and 2.66). Therefore, there were six different thresholds in total used to identify and analyze iMEPs [4–7, 18–21, 23–25]. The SD method uses thresholds based on the average rectified pre-TMS EMG, whereas the MCD method uses thresholds based on the variability in the rectified pre-TMS EMG. IMEP onset was defined as the time point at which the rectified post-TMS EMG exceeded the pre-TMS EMG by threshold level for at least 5 ms and iMEP offset was defined as the time point at which EMG returns below this level for 50% of the data points in the following 10 ms window [21]. IMEP data were only used for analysis if the iMEP onset was present in the 5–40 ms time window following the TMS artifact, resulting in iMEP analysis as a bounded estimate for other features. IMEP amplitude and area were calculated as in the subjective method.

In addition to the objective methods described above, we also explored the reliability of the recently published cross-correlation method for iMEP detection (trial-by-trial analysis) [14]. We modified the cross-correlation method to compute the correlation between the average post-TMS EMG signal and individual post-TMS EMG signal from a participant. We classified the individual post-TMS signal as iMEP if the correlation was above 0.7.

#### 2.3.3. Inter-rater reliability

The degree of agreement between the two raters for iMEP detection was determined using the percent agreement [31]. The degree of agreement between the two raters for analyzing iMEP features (peak-to-peak amplitude, area, onset, and offset) was determined using the intraclass correlation coefficient (ICC, two-way, absolute agreement) [31, 32]. ICC values less than 0.5 indicate poor reliability, values between 0.5 and 0.75 indicate moderate reliability, values between 0.75 and 0.9 indicate good reliability and values greater than 0.90 indicate excellent reliability.

#### 2.3.4. Comparison of subjective vs. objective methods

We compared the objective method against the subjective method to determine a reliable, objective method (threshold level) that exhibits the highest agreement with the subjective method. For iMEP detection, we selected the trials mutually agreed upon by both raters in the trial-by-trial and average-signal analysis approaches. Trials with disagreements were further reviewed by a third rater (EBP). Subjective and objective methods for detecting iMEPs were compared using the percent agreement. For iMEP features (peak-to-peak amplitude, area, onset, and offset), we have only included trials selected by the raters for comparison with the objective methods (identical trials), and iMEP negative trials were excluded from the analysis. We averaged the values of rater-selected iMEP trials in both trial-by-trial and average-signal analysis approaches, the grand average values were compared against the objective methods for iMEP analysis. Subjective and objective methods for analyzing iMEP features were then compared using the intraclass correlation coefficient (ICC, two-way, absolute agreement). For iMEP onset and offset, we also compared the difference between the average value of rater-selected iMEP trials with algorithm-selected iMEP values in both trial-by-trial and average-signal analysis approaches. We used the median and interquartile range values of the differences (Δ) to determine if the differences were clinically significant (Δ ⩾ 3 ms). All analyses were performed using the R programming language (R 4.0.0 for Windows) [33].

## 3. Results

Thirty-two chronic stroke participants (17 female, 15 male) with mean age of 60.9 (SD 8.1) years and average time since stroke of 27.1 (SD 20.4) months were enrolled. The mean UEFM score was 21.3 (SD 8.8) and UL impairment ranged from mild to severe (UEFM range: 11–43) [34]. Of the 32 participants, only 9 had cMEPs on the paretic EDC muscle, which indicates significant damage to the crossed pathways originating from the contralateral motor cortex in at least 72% of the participants. Clinico-demographic characteristics of the participants are shown in table 1.

**Table 1.** Demographic characteristics of participants with upper limb paresis.

Subject ID	Age/ Gender	Race	Affected Side	Dominant Paresis	Stroke Type	Months Since Stroke	UE Fugl Meyer Total (Max 66)	Contralateral MEP ±
1	60/F	White	L	N	Hemorrhagic	29	19	—
2	61/M	White	R	Y	Ischemic	69	41	+
3	75/F	White	R	Y	Ischemic	27	17	—
4	52/M	White	L	N	Ischemic	46	13	—
5	68/F	White	R	Y	Ischemic	29	17	—
6	60/F	Black/ African American	R	Y	Ischemic	29	28	+
7	55/F	White	L	Y	Ischemic	11.9	21	—
8	53/M	White	R	Y	Hemorrhagic	6.2	16	—
9	64/M	White	L	N	Hemorrhagic	11.3	19	—
10	58/F	Black/ African American	R	Y	Ischemic	17.2	33	—
11	54/M	Black/ African American	L	N	Hemorrhagic	22.6	22	—
12	67/F	White	R	Y	Ischemic	16.4	20	—
13	76/F	White	R	N	Ischemic	15.2	29	+
14	62/M	White	R	Y	Ischemic	15.9	35	+
15	49/F	Unknown	R	Y	Ischemic	6.6	39	—
16	50/M	White	L	Y	Ischemic	7	27	—
17	66/F	White	L	N	Ischemic	6	43	—
18	60/M	Black/ African American	R	Y	Ischemic	68	18	—
19	52/F	White	L	N	Ischemic	51	19	+
20	55/M	White	L	N	Ischemic	48	13	—
21	57/F	White	L	N	Ischemic	12	13	—
22	55/F	White	L	N	Ischemic	20	14	—
23	83/M	Black/ African American	R	N	Ischemic	11	19	+
24	56/M	White	L	N	Ischemic	10	11	—
25	75/F	White	R	Y	Ischemic	12	15	—
26	57/F	White	R	Y	Hemorrhagic	71	23	+
27	52/F	Black/ African American	R	Y	Ischemic	16	12	—
28	62/M	Black/ African American	L	Y	Ischemic	38	11	+
29	61/F	White	R	Y	Ischemic	11	22	—
30	65/M	Black/ African American	L	N	Ischemic	69	19	—
31	63/M	White	R	Y	Ischemic	26	19	—
32	65/M	Black/ African American	L	N	Ischemic	40	13	+

**Mean (SD)**	**60.9 (8.1)**					**27.1 (20.4)**	**21.3 (8.8)**	**9(+)**
**Count**	**15 M**	**22 (White)**	**15(L)**	**18(Y)**	**27 (Ischemic)**			
	**17 F**	**9 (Black/ African American**			**5 (Hemorrhagic)**			

### 3.1. Inter-rater reliability

The degree of agreement between the two raters for detecting iMEPs was above 85% for both trial-by-trial and average-signal approaches (table 2). Inter-rater agreement was excellent (ICC > 0.9) for iMEP amplitude and area in both trail-by-trail and average-signal analysis approaches. Onset of iMEPs, however, showed differing levels of inter-rater agreement depending on which approach was used. Rater agreement was good (ICC = 0.83) when iMEP onset was detected on a trial-by-trial analysis approach, whereas rater agreement was moderate (ICC = 0.52) when iMEP onset was detected on an average-signal analysis approach. Nevertheless, the differences (median ± interquartile range (ms)) between the raters were small with respect to iMEP onset of 1.0 ms (±2.3 ms) in the trial-by-trial analysis and 0.8 ms (±1.4 ms) in the average-signal analysis. Offset of iMEPs also showed differing levels of inter-rater agreement depending on which approach was used. Rater agreement was good (ICC = 0.83) when iMEP offset had to be detected on a trial-by-trial analysis approach, whereas rater agreement was excellent (ICC = 0.93) when iMEP offset had to be detected on an average-signal analysis approach. The differences (median ± interquartile range (ms)) between raters were small with respect to iMEP offset of 1.5 ms (±2.1 ms) in the trial-by-trial analysis and 1.0 ms (±1.4 ms) in the average-signal analysis.

**Table 2.** Inter-rater reliability of subjective (rater-based) method for iMEP analysis.

Feature	Trial-by-trial approach	Average-signal approach
iMEP detection (Percent agreement)	85.6%	87.5%
iMEP amplitude (ICC)	0.99 (Excellent)	0.99 (Excellent)
iMEP area (ICC)	0.99 (Excellent)	0.99 (Excellent)
iMEP onset (ICC)	0.83 (Good)	0.52 (Moderate)
iMEP offset (ICC)	0.83 (Good)	0.93 (Excellent)

ICC—Intraclass correlation.

### 3.2. Agreement between subjective vs. Objective methods

#### 3.2.1. Trial-by-trial analysis

**Figure 3.**
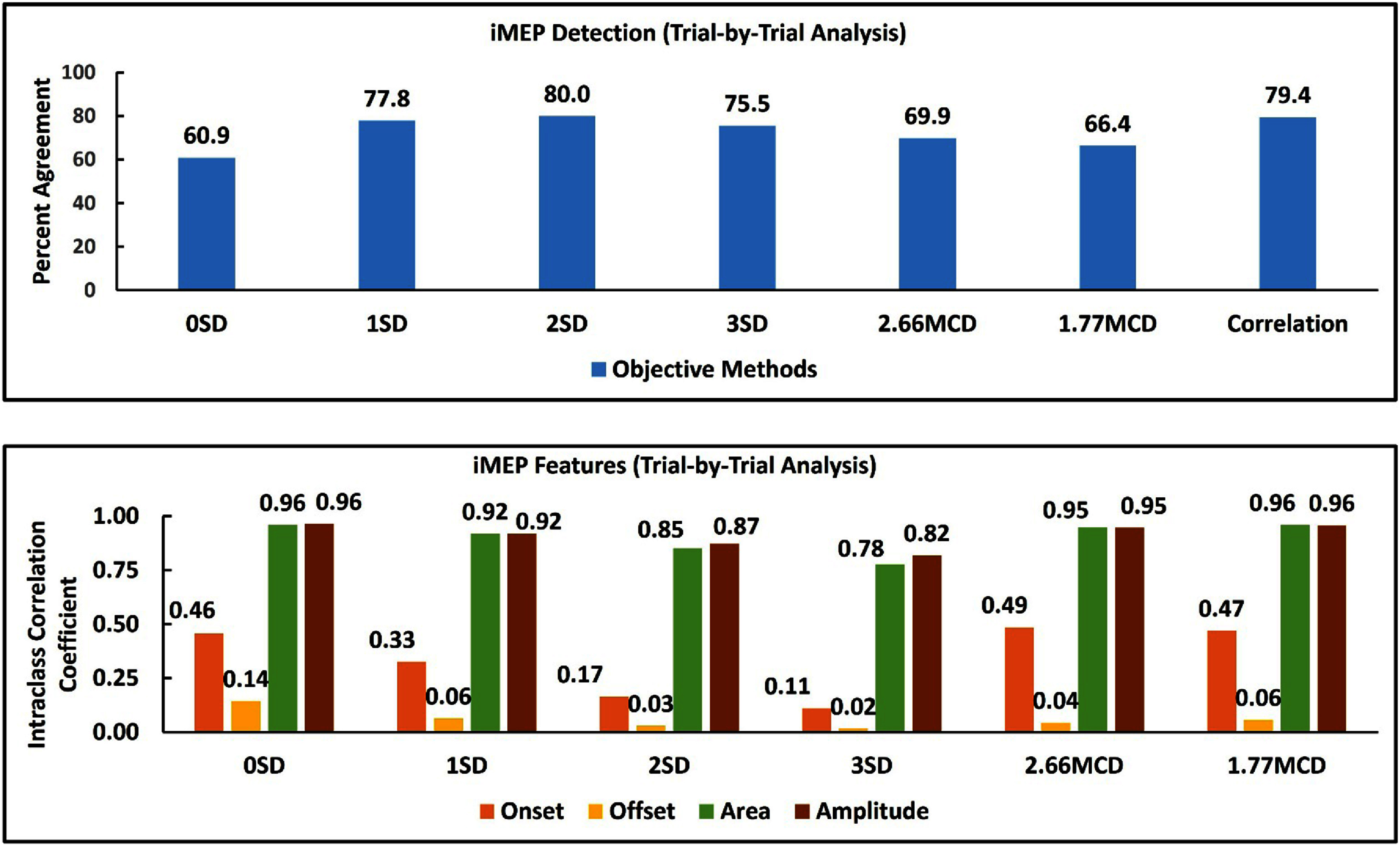
Agreement between subjective and objective methods for iMEP features (presence/absence, amplitude, area, onset, and offset) in trial-by-trial analysis.

The agreement between subjective and objective methods for detecting iMEPs was over 75% for 1SD, 2SD, 3SD, and Correlation-based methods (figure 3). The number of iMEPs detected by each objective method is also shown in table 3. The agreement between the subjective and objective methods for analyzing iMEP amplitude and area was excellent (ICC > 0.9) for 0SD, 1SD, 2.66MCD, and 1.77MCD methods. The agreement between the subjective and objective methods for analyzing iMEP onset and offset was poor (ICC < 0.5). The differences (median ± interquartile range (ms)) between the subjective and objective methods were large (low reliability, not clinically significant) in the trial-by-trial analysis approach (table 4).

**Table 3.** Comparison of subjective and objective methods for iMEP detection in trial-by-trial analysis.

Method	Number of iMEPs Identified (Total 1225 trials)
Subjective method	378
0SD method	679
1SD method	452
2SD method	213
3SD method	100
2.66MCD method	595
1.77MCD method	620
Correlation method	338

**Table 4.** Comparison of subjective and objective methods for analyzing iMEP onset and offset in trial-by-trial analysis.

Feature	Rater—0SD median (IQR)	Rater—1SD median (IQR)	Rater—2SD median (IQR)	Rater—3SD median (IQR)	Rater—2.66MCD median (IQR)	Rater—1.77MCD median (IQR)
iMEP onset difference (ms)	2.7 (7.8)	4.8 (10.4)	12.1 (14.4)	15.6 (10.2)	2.9 (7.4)	2.8 (7.4)
iMEP offset difference (ms)	3.5 (8.1)	6.5 (16.8)	24.9 (28.8)	32.8 (19.3)	4.3 (9.9)	4.0 (10.1)

IQR—interquartile range.

#### 3.2.2. Average-signal analysis

**Figure 4.**
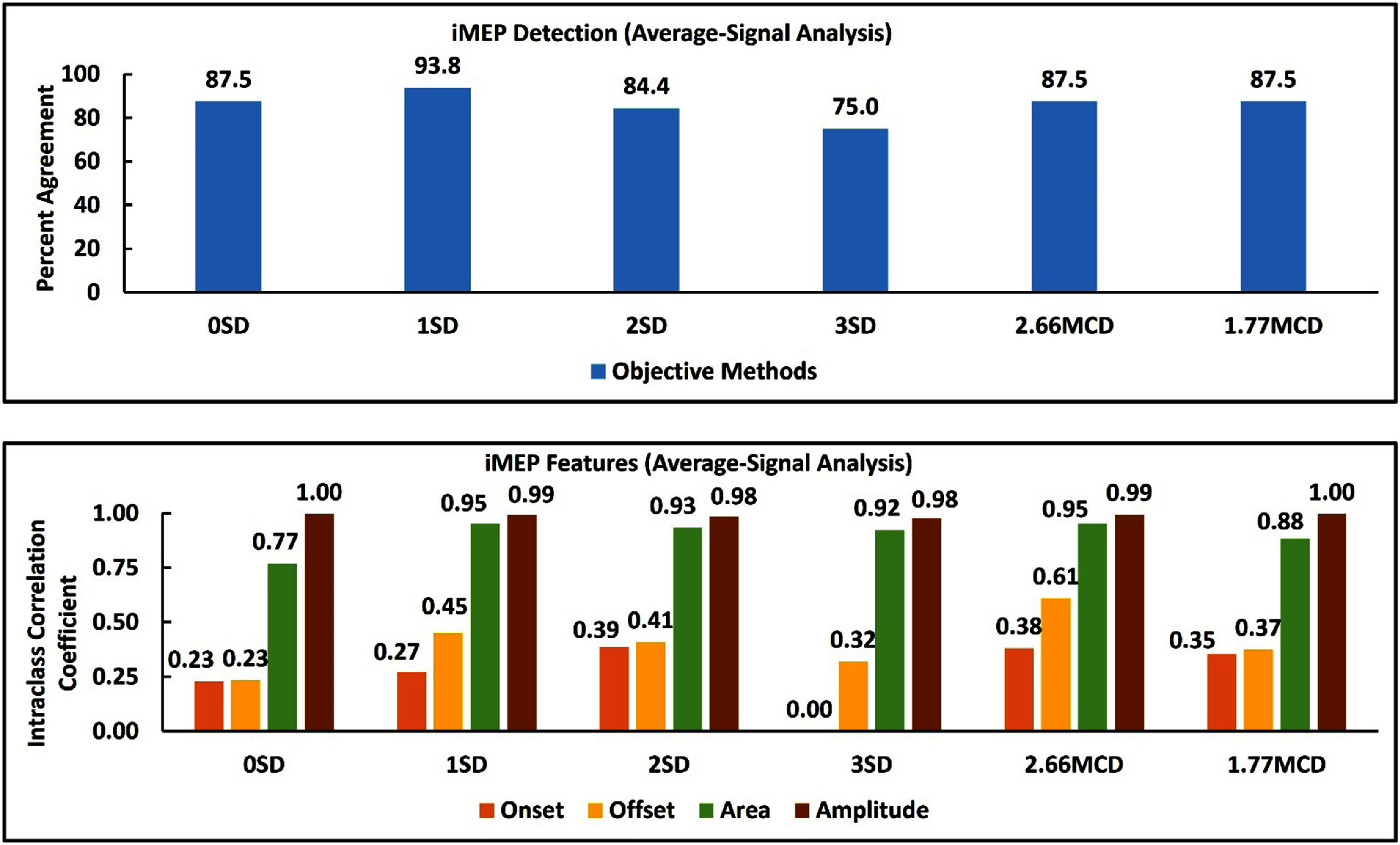
Agreement between subjective and objective methods for iMEP features (presence/absence, amplitude, area, onset, and offset) in average-signal analysis.

The agreement between the subjective and objective methods for identifying iMEPs was over 90% for the 1SD method (figure 4). The number of iMEPs detected by each objective method is also shown in table 5. The agreement between the subjective and objective methods for analyzing iMEP amplitude and area was excellent (ICC > 0.9) for 1SD, 2SD, 3SD, and 2.66MCD methods. The agreement between the subjective and objective methods for analyzing iMEP onset was poor (ICC < 0.5). The differences (median ± interquartile range (ms)) between the subjective and objective methods for iMEP onset were also large (low reliability, not clinically significant) in the average-signal analysis approach (table 6). The agreement between the subjective and objective methods for analyzing iMEP offset was moderate (ICC = 0.61) for the 2.66MCD method. The difference (median ± interquartile range (ms)) between the subjective and 2.66 MCD method for iMEP offset was also small in the average-signal analysis approach (table 6).

**Table 5.** Comparison of subjective and objective methods for iMEP detection in average-signal analysis.

Method	Number of iMEPs identified (Total 32 trials)
Subjective method	27
0SD method	31
1SD method	27
2SD method	22
3SD method	19
2.66MCD method	31
1.77MCD method	31

**Table 6.** Comparison of subjective and objective approaches for iMEP onset and offset in the average-signal analysis.

Feature	Rater—0SD median (IQR)	Rater—1SD median (IQR)	Rater—2SD median (IQR)	Rater—3SD median (IQR)	Rater—2.66MCD median (IQR)	Rater—1.77MCD median (IQR)
iMEP onset difference (ms)	3.0 (9.5)	2.1 (8.6)	3.8 (8.1)	7.3 (13.3)	2.1 (7.9)	2.1 (8.6)
iMEP offset difference (ms)	1.4 (2.8)	3.8 (5.4)	2.5 (19.8)	6.5 (19.5)	1.6 (3.6)	1.6 (3.1)

IQR—interquartile range.

## 4. Discussion

This study sought to investigate inter-rater reliability of iMEP features collected from the paretic proximal forearm flexor muscle (biceps brachii) in a sample of 32 stroke survivors with chronic upper limb paresis. We also examined the agreement between subjective (rater-determined) and objective (algorithm-driven) methods of iMEP analysis. Our findings reveal an excellent agreement between the two raters for iMEP detection, amplitude, and area. Of all the objective methods we tested, the 1SD method was the best for identifying and analyzing iMEP amplitude and area in both trial-by-trial and average-signal analysis approaches. Results from this study also reveal that the 1SD-based average-signal analysis approach is best suited for identifying iMEPs and analyzing iMEP area and amplitude compared to the trial-by-trial analysis approach. Upper limb rehabilitation studies designed to understand the role of ipsilateral mechanisms in stroke motor recovery can benefit from our systematic, patient-specific analysis approaches presented in the study.

SD based analysis approaches were commonly reported for the identification and analysis of iMEPs [4–7, 18–20, 24, 25]. Results from our study support the previous analysis approach, showing that the 1SD method was most appropriate for identifying and analyzing iMEP amplitude and area in stroke survivors with chronic UE motor impairment. Prior research observed an inverse relationship between strictness of algorithms (i.e. the 3SD approach is stricter than 2SD for analyzing iMEPs) and reliability [14]. Results from our study show that the 1SD threshold is the optimal criterion for identifying and analyzing the weak iMEP signal in the presence of ongoing paretic muscle contraction. Neurophysiological studies where the evoked potential has a low signal-noise ratio (e.g. EEG event-related potentials) support using average-signal analysis approaches [35]. Results from our study also support using the average-signal analysis approach over the trial-by-trial analysis approach, showing that the 1SD-based average-signal analysis approach is best suited for identifying iMEPs (>90% agreement) and analyzing iMEP area and amplitude (ICC > 0.9, excellent reliability). However, it could be useful to analyze iMEPs in the trial-by-trial analysis approaches to understand the iMEP prevalence in the patient population. The neurophysiological relevance of both analysis approaches needs to be determined in future studies.

None of the objective methods were reliable for analyzing iMEP onset and offset. Our findings range from an error as low as 2.1 ms to as high as 15.6 ms (see tables 4 and 6). Still, it is unclear what is an acceptable error to make meaningful interpretations of changes in iMEP latency. The lack of agreement between subjective and objective data for iMEP onset and offset may be contributed by the intra-rater variability, the inability of objective methods to precisely identify the onset and offset from the noisy background, or the small sample size of our study. To improve onset and offset accuracy, it will be important to translate recent automated, adaptive methods for identifying cMEP onset and offset to investigate whether these automated methods improve the reliability of iMEP latency analysis in individuals with chronic post-stroke hemiparesis [36, 37]. Although the onset and offset of iMEP could not be reliably measured using objective methods, this did not compromise the reliability of other iMEP features, such as detection, amplitude, and area. From a clinical perspective, accurate iMEP detection is promising for guiding treatment decisions targeting the ipsilateral pathways—such as the reticulospinal, rubrospinal, and vestibulospinal tracts. Additionally, iMEP amplitude and area could provide valuable insights for future neurophysiological studies of these pathways [7].

## 5. Conclusions

Our study reports excellent inter-rater reliability for identifying and analyzing iMEP amplitude and area in both trial-by-trial and average-signal analysis approaches for individuals with chronic post-stroke hemiparesis. Agreement between subjective (rater-determined) and objective (algorithm-driven) iMEP analysis methods shows that the 1SD objective method is most suitable for identifying and analyzing these difficult-to-read ipsilateral pathway potentials in both trial-by-trial and average-signal analysis approaches. Our findings indicate that an objective algorithmic approach can provide precision to the subjective method and minimize the human effort and time required for performing the iMEP analysis.

## Data Availability

The data cannot be made publicly available upon publication because they are owned by a third party and the terms of use prevent public distribution. The data that support the findings of this study are available upon reasonable request from the authors.
